# Aging Men’s and Women’s Religious Identities: Straightforward or Complex?

**DOI:** 10.1093/geroni/igaf122.3127

**Published:** 2025-12-31

**Authors:** Kyra Hultgren, Sarah MacDougall, Sophia Paleski, Alessia Petroni, Edward Thompson, Christopher Turner, Michiko Iwasaki, Andrew Futterman

**Affiliations:** Loyola University Maryland, Baltimore, Maryland, United States; Loyola University Maryland, Baltimore, Maryland, United States; Loyola University Maryland, Baltimore, Maryland, United States; Loyola University Maryland, Baltimore, Maryland, United States; College of the Holy Cross, Worcester, Massachusetts, United States; Loyola University Maryland, Baltimore, Maryland, United States; Loyola University Maryland, Baltimore, Maryland, United States; Loyola University Maryland, Baltimore, Maryland, United States

## Abstract

This study explores the determinants of older adults taking on a “straightforward” or a “complex” orientation to religious belief and behavior. Two hundred and seventy-eight semi-structured interviews of older individuals describing their religious belief and behavior, their religious doubts, and their religious development were used. From these, 40 interviews were selected as either prototypically “straightforward” or “complex” (20 each). Complexity was defined primarily in cognitive and emotional terms as a form of post-formal reasoning and reflective emotionality (Labouvie-Vief, 2000) where religious doubt and faith coexist and individuals describe their religious life as involving a “reflective component” (Labouvie-Vief, 2015). By contrast, religious straightforwardness is defined by individuals who demonstrate either religious faith or doubt, but not both, without extensive reflection. Identification of interviews demonstrating prototypically “straightforward” and “complex” religious belief and behavior was first agreed upon by all researchers, and then the reasons for religious straightforwardness and complexity were assessed. Initial analyses indicated that life stressors, i.e., events beyond one’s control (Labouvie-Vief, 2015), were primarily the reason for increased religious straightforwardness. Individuals with “complex” religiousness described little vulnerability and dependence, and an openness to new experience. Other factors described as important reasons for straightforwardness were being above 80-85 years of age (age range 60-101 years), membership in a “conservative” denominational family, and being male. We compare these results to results from our previous quantitative study of religious complexity using a random sample of older adults and discuss our findings in terms of theories of age-related cognitive and emotional complexity.

